# Retrospective DVH analysis of point A based intracavitary brachytherapy for uterine cervical cancer

**DOI:** 10.1093/jrr/rrz099

**Published:** 2020-02-01

**Authors:** Masanori Someya, Tomokazu Hasegawa, Takaaki Tsuchiya, Mio Kitagawa, Toshio Gocho, Yuuki Fukushima, Masakazu Hori, Katsutoshi Miura, Yu Takada, Kensei Nakata, Koh-ichi Sakata

**Affiliations:** 1 Department of Radiology, Sapporo Medical University School of Medicine, Chuo-ku, S1 W16, Sapporo, Hokkaido 060-8543, Japan; 2 Department of Radiology, Sapporo City General Hospital, Hokkaido, Japan; 3 Department of Radiation Oncology, Teine Keijinkai Hospital, Sapporo, Hokkaido, Japan

**Keywords:** cervical cancer, radiotherapy, brachytherapy, DVH analysis, high-risk CTV

## Abstract

Combining external beam radiotherapy (EBRT) with intracavitary brachytherapy (ICBT) is important for definitive treatment of cervical cancer. In cervical cancer patients receiving radiotherapy, we evaluated treatment outcomes in relation to dose–volume histogram parameters, including the computed tomography (CT)-based high-risk clinical target volume (HR-CTV) for ICBT. Between 2010 and 2015, 89 consecutive cervical cancer patients were mostly treated with 40 Gy of EBRT in 20 fractions and 18 Gy of ICBT prescribed to point A in 3 fractions. CT scans were obtained during ICBT. The HR-CTV D90 was calculated and the total doses of ICBT and EBRT were converted to the equivalent dose in 2 Gy fractions (EQD2). When the patients were divided into four groups according to EQD2 of the HR-CTV D90, the 3-year local recurrence-free survival rates were 95.2, 78.4, 52.7 and 42.9% for patients receiving >80 , 70–80 , 60–70 and <60 Gy, respectively. There was a significant negative correlation between EQD2 of the HR-CTV D90 and the HR-CTV volume at first ICBT (*r* = −0.713). Local recurrence was more frequent when the HR-CTV volume was ≥22 cc and EQD2 of the HR-CTV D90 was <70 Gy. Multivariate analysis showed that EQD2 of the HR-CTV D90 and concurrent chemotherapy (≥4 cycles) were significant determinants of overall survival. HR-CTV D90 was an important prognostic indicator for local recurrence. HR-CTV D90 >70 Gy is required for the better local control, especially in patients with a larger HR-CTV (≥22 cc at initial ICBT).

## INTRODUCTION

It is important to combine external beam radiotherapy (EBRT) with intracavitary brachytherapy (ICBT) for definitive treatment of cervical cancer, and concurrent chemoradiotherapy (CCRT) is recommended for patients with advanced cancer.

In contrast to standard 2D treatment planning for ICBT based on point A, individualized treatment planning has recently been performed for 3D image guided brachytherapy (3D-IGBT). 3D-IGBT planning is based on 3D imaging modalities such as computed tomography (CT) combined with magnetic resonance imaging (MRI), and is performed with the applicators inserted. Appropriate contouring of both the target volume and organs at risk (OAR) is essential for appropriate delivery of 3D-IGBT. It is important to determine that the high-risk clinical target volume (HR-CTV) since it serves as a reference for both prescription and evaluation of 3D-IGBT.

In patients receiving MRI-based IGBT, dose–effect relationships have been demonstrated for the local control rate as well as the incidence and severity of complications [1–4]. Murakami *et al*. also reported similar results for a series of patients receiving CT-based IGBT. These reports suggest that HR-CTV D90 is one of the most important predictors of local control [5]. To minimize variations of CT-based HR-CTV contouring and differences among physicians, consensus-based guidelines were recently developed by the Gynecological Tumor Committee Members of the Japanese Radiation Oncology Study Group (JROSG) [6].

At our institution, we have performed 2D-based ICBT based on point A and have obtained CT scans after treatment, mainly to check whether insertion of the applicator was adequate. In this study, we retrospectively evaluated treatment outcomes and late complications, including the dose–volume histogram (DVH) parameters of ICBT in cervical cancer patients receiving radiotherapy. For dose–response analyses, we used the CT-based definition of HR-CTV proposed by the JROSG.

## MATERIALS AND METHODS

Between May 2010 and August 2015, a total of 89 consecutive patients with stage I–IVa cervical cancer underwent ICBT at our hospital. In 44 of these patients, EBRT was performed at four other hospitals and they were referred to us for ICBT, while the other 45 patients received both EBRT and ICBT at our hospital. Patient characteristics are listed in Table 1.

**Table 1:** Patient characteristics; median values are shown in parentheses

			*n* = 89
Age			31–88 (62)
Pathology			
	SCC		82
	ASC		2
	AC		5
FIGO stage (2008)			
	Ia		1
	Ib1		5
Ib2			1
	IIa		2
	IIb		35
	IIIa		3
	IIIb		35
	IVa		7
Pelvic lymph node metastasis			
	No		32
Yes			57
Pretreatment tumor diameter (cm)			1.5–11 (5.2)
Pretreatment tumor volume (cc)			10.0–247.8 (60.2)
Measured by MR		76	
CT		13	
Pretreatment hemoglobin level (g/dl)			6.2–15.4 (11.7)
Pretreatment SCC antigen level (ng/ml)			0.8–194.0 (6.9)

SCC = Squamous cell carcinoma, ASC = adenosquamous cell carcinoma, AC = adenocarcinoma.

Before treatment, all 89 patients had chest–abdomen–pelvis CT scans and 76 patients underwent pelvic MRI. In addition, pelvic MRI was performed within 1 week prior to the first ICBT session in all patients.

Treatment characteristics are shown in Table 2. EBRT was delivered by the 3D conformal technique using a linear accelerator with a 10 MV photon beam. The initial dose of 30–50 Gy (in 15–25 fractions) was delivered to the whole pelvis (WP) using a 4-field box technique. Subsequently, 81 patients received 4–20 Gy (in 2–10 fractions), delivered to the WP with 2–5 cm of central shielding (CS) depending on the size of the primary tumor and the International Federation of Gynecology and Obstetrics (FIGO) stage.

**Table 2:** Treatment characteristics; median values are shown in parentheses

EBRT dose (whole pelvis) + ICBT point A dose				
30–30.6 Gy/15–17 fr	20.4–24 Gy/4 fr			11 (11.3%)
39.6–40 Gy/20–22 fr	17.3 Gy/2 fr			5 (5.6%)
	17.3–18 Gy/3 fr			51 (57.3%)
50–50.4 Gy/25–28 fr	12–14.5 Gy/2–3 fr			22 (27.8%)
Width of CS				
2 cm			1 (1.3%)	
3 cm			11 (13.6%)	
4 cm			67 (82.7%)	
5 cm			2 (2.5%)	
Dose of CS (Gy)			3.6–22 (12.0)	
Volume of HR-CTV (cc) at initial ICBT			10.0–108.7 (18.2)	
Total EQD2 (whole pelvic EBRT + ICBT, Gy)				
HR-CTV D90			50.4–109.6 (69.8)	
Rectal D2cc			48.7–142.7 (69.9)	
Bladder D2cc			52.8–121.7 (74.7)	
OTT (days)				36–83 (54)
Neoadjuvant chemotherapy				4 (4.5%)
Concurrent chemotherapy				62 (69.7%)
Weekly CDDP			28 (45.2%)	
1–3 course		14 (50.0%)		
4–6 course		14 (50.0%)		
Weekly nedaplatin			34 (54.8%)	
1–3 course		7 (20.6%)		
4–6 course		27 (79.4%)		
Adjuvant chemotherapy				28 (31.4%)

EBRT = External beam irradiation, ICBT = Intracavitary brachytherapy, CS = Central Shielding, HR-CTV = High risk CTV, OTT = Overall treatment time.

In 85 patients, ICBT was performed using tandem with ovoid applicators (Asian Pacific type, 83; Fletcher Williamson, 2), while a vaginal cylinder was used in 4 patients. ICBT was delivered with a high-dose rate (HDR) ^192^Ir remote afterloading system (RALS) (microSelectron, Elekta, Stockholm, Sweden). Treatment planning was based on 2-D X-ray films according to the Manchester system. CT scans with a 2.5 mm slice thickness were obtained after treatment just before removal of applicators. The dose delivered to point A was calculated in all patients. Most patients received 40 Gy of EBRT in 20 fractions and 18 Gy of ICBT in 3 fractions. After installation of the HDR ^192^Ir RALS system, we followed our protocol for the previous HDR ^60^Co-RALS system from May 2010 to Oct 2010. As a result, 5 patients received 17.3 Gy of ICBT in 2 fractions before we switched to the current ICBT protocol.

ICBT was performed once a week. EBRT and ICBT were not given on the same day. All planning for actual ICBT treatment was done with Oncentra ver. 4.3 (Nucletron, Veenendaal, The Netherlands). For this study, two radiation oncologists (T.H. and M.S.) used MRI images obtained before treatment and before ICBT to retrospectively review the OAR and for delineation of the HR-CTV according to the JROSG guidelines, based on mutual consensus [6]. The HR-CTV volume and the distance from the tandem to the border of the HR-CTV were measured on CT images of initial ICBT.

Dose parameters were calculated for each session of ICBT, including the dose at point A, the minimum dose to the most irradiated 2 cc (D2cc) to the OARs and HR-CTV D90. The total doses delivered during ICBT and WP-EBRT converted to EQD2 were calculated using a linear–quadratic model, with α/β = 10 Gy for the tumors and α/β = 3 Gy for OAR. The CS dose was not included in this calculation. The distance from the tandem to the lateral border of the HR-CTV was also measured with Oncentra ver. 4.3.

Four patients received three courses of neoadjuvant chemotherapy (TC therapy with carboplatin and paclitaxel). Sixty-two patients received CCRT, which involved weekly administration of cisplatin or nedaplatin at a dose of 30–40 mg/m^2^ together with radiotherapy (Table 2). Among them, 14/28 patients (50%) receiving Cisplatin (CDDP) and 27/34 patients (79.4%) receiving nedaplatin (NDP) completed at least 4 cycles of concurrent chemotherapy. Twenty-eight patients were given adjuvant chemotherapy at their gynecologist’s discretion, which was 3–6 cycles of either chemotherapy with carboplatin and paclitaxe (TC) therapy or NDP and irinotecan (NDP/CPT) therapy.

After completing treatment, patients were followed up every 1–3 months by radiation oncologists and gynecologists. At each follow-up visit, they underwent a pelvic examination and laboratory tests, including measurement of tumor markers [squamous cell carcinoma (SCC), Carcinoembryonic antigen (CEA), CA125 and CA19–9]. CT scans of the abdomen and pelvis and chest X-rays (or CT scans) were obtained every 6–12 months.

Adverse events affecting the bladder, rectum, and small and large intestines were evaluated according to the National Cancer Institute Common Toxicity Criteria, version 4.0 (CTCAE 4.0). The median follow-up time was 46 months (range, 6–108 months). Among the surviving patients, 2 were lost to follow-up within 3 years (at 12 and 26 months). The cut-off date for this analysis was April 2019.

Survival was estimated by the Kaplan-Meier method and the significance of differences in survival was determined by the log-rank test. Cox proportional hazards analysis was used to identify variables that influenced oncologic outcomes. Stepwise multivariate analysis was performed with a cut-off *P*-value of <0.1. All statistical analyses were done with BellCurve for Excel 2.00 (BellCurve, Tokyo, Japan).

This study was approved by our institutional review board (Approval No. 292–36).

## RESULTS

At the time of analysis, 6 of the 89 patients had residual disease after initial treatment and 35 patients had recurrence. Of these 35 recurrent patients, 17 patients had recurrence confined to the pelvis (cervix in 15 and pelvic lymph nodes in 2), 12 patients had distant metastasis alone, and 6 patients had both pelvic recurrence and distant metastasis.

Thirty-four patients died of cervical cancer, 2 patients died of other cancers, 5 died of intercurrent disease, and 1 patient was alive with disease. The overall treatment time (OTT) ranged from 36 to 83 days, with a median of 54 days. OTT was >63 days in 6 patients, and their details are displayed in Table 3.

**Table 3:** Details of patients whose overall treatment times were >63 days

No.	Age (years)	FIGO stage	Tumor size (cm)	Histology	OTT (days)	EQD2 HR-CTV D90 (Gy)	Concurrent chemotherapy	Reason for delay	Outcome
1.	86	IIB	3.9	SCC	77	77.0	None	1 Month delay due to dislocation of artificial femoral head	Local recurrence 19 months
2.	62	IIIb	5.7	SCC	64	86.3	wNDP^*^4	National holiday poor general condition	Alive 40 months
3.	43	IIIa	6.5	SCC	64	58.1	wNDP^*^6	1 Week delay due to Tandem perforation at 1st RALS	Pelvic LN rec 12 months
4.	56	IIIb	5.6	SCC	83	60.2	wNDP^*^5	1 Month delay due to ileus from fallopian tube abscess	Local recurrence 4 months
5.	72	IIIb	7.1	SCC	65	74.2	wNDP^*^4	1 Week delay from EBRT to ICBT and national holiday	Alive 64 months
6.	52	IIIb	4.3	SCC	68	66.7	wCDDP^*^3	3 Week delay due to grade 3 leukocytepenia	Distant metastasis 6 months

wNDP^*^4,5,6 = 4, 5, or 6 cycles of weekly nedaplatin administration, LN rec = lymph node recurrence, wCDDP^*^3 = 3 cycles of weekly Cisplatin administration.

**Fig. 1.**
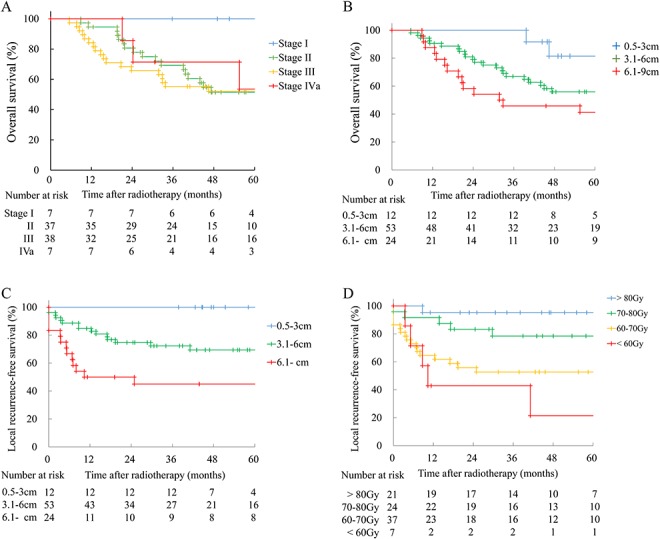
Overall survival and local recurrence-free survival rates. (**A**) Overall survival according to FIGO stage. Numbers at risk are shown below the Kaplan-Meier curves. (**B**) Overall survival stratified by pretreatment tumor diameter. (**C**) Local recurrence-free survival stratified by pretreatment tumor diameter. (**D**) Local recurrence-free survival stratified by EQD2 of the HR-CTV D90.

Figure 1A shows overall survival according to FIGO stage. The 3-year overall survival rates were 100, 69.3, 55.3, 71.4 and 42.3% for patients in stages I, II, III, IVa and IVb, respectively. Figure 1B displays overall survival stratified by the pretreatment tumor diameter. When the patients were divided into three groups according to tumor diameter, 3-year overall survival was 100, 67.0, and 45.8% for patients with tumors measuring 0.5–3, 3.1–6 and ≥6.1 cm, respectively. Local recurrence-free survival stratified by the pretreatment tumor diameter is shown in Figure 1C. When patients were again divided into three groups according to tumor diameter, 3-year local recurrence-free survival was 100, 72.3 and 45.0% for patients with tumors measuring 0.5–3, 3.1–6 and ≥6.1 cm, respectively. Local recurrence-free survival stratified by EQD2 of the HR-CTV is displayed in Figure 1D. When we divided the patients into four groups according to EQD2, 3-year local recurrence-free survival was 95.2, 78.4, 52.7 and 42.9% for patients receiving >80 Gy, 70–80 Gy, 60–70 Gy and <60 Gy, respectively.

**Fig. 2.**
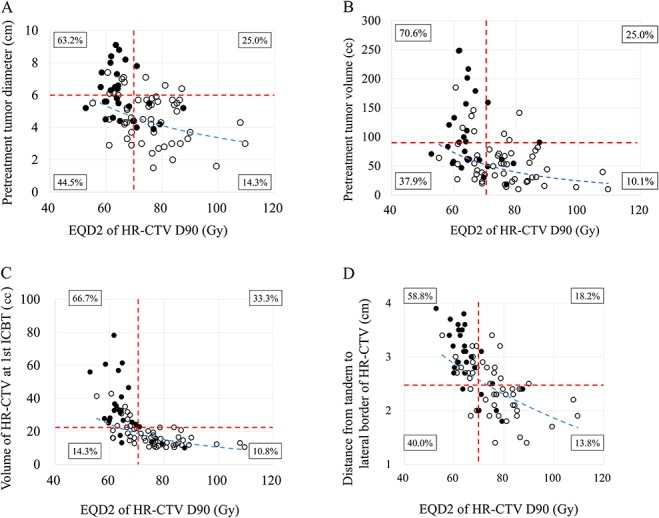
(**A**) Relationship between EQD2 of the HR-CTV D90 and pretreatment tumor diameter. Closed circles represent patients with local recurrence and open circles indicate patients without recurrence. Red dotted lines show cut-off values of 6 cm (horizontal) and 70 Gy (vertical). The blue dotted line is the regression curve. There was a significant negative correlation between EQD2 of the HR-CTV D90 and pretreatment tumor diameter (*r* = −0.459, *P* < 0.001). Percentages in boxes indicate the local recurrence rate for each quadrant. (**B**) Relationship between EQD2 of the HR-CTV D90 and pretreatment tumor volume. Closed circles represent patients with local recurrence and open circles indicate patients without recurrence. Red dotted lines show cut-off values of 90 cc (horizontal) and 70 Gy (vertical). The blue dotted line is the regression curve. There was a significant negative correlation between EQD2 of the HR-CTV D90 and pretreatment tumor volume (*r* = −0.513, *P* < 0.001). Percentages in boxes indicate the local recurrence rate for each quadrant. (**C**) Relationship between EQD2 of the HR-CTV D90 and the HR-CTV volume at first ICBT. Closed circles indicate patients with local recurrence and open circles represent patients without recurrence. Red dotted lines show cut-off values of 22 cc (horizontal) and 70 Gy (vertical). The blue dotted line is the regression curve. A significant negative correlation was noted between EQD2 of the HR-CTV D90 and the HR-CTV volume at first ICBT (*r* = −0.713, *P* < 0.001). Percentages in boxes indicate the local recurrence rate for each quadrant. (**D**)Relationship between EQD2 of the HR-CTV D90 and the distance from tandem to the lateral border of the HR-CTV. Closed circles represent patients with local recurrence and open circles are for patients without recurrence. Red dotted lines indicate cut-off values of 2.5 cm (horizontal) and 70 Gy (vertical). The blue dotted line shows the regression curve. A negative correlation was recognized between EQD2 of the HR-CTV D90 and the distance from tandem to the lateral border of the HR-CTV (*r* = − 0.685, *P* < 0.001). Percentages in boxes indicate the local recurrence rate for each quadrant.

Figure 2A reveals the relationship between EQD2 of the HR-CTV D90 and pretreatment tumor diameter is shown. Local recurrence was more frequent when the tumor diameter was >6 cm and EQD2 was <70 Gy. There was a strong correlation between pretreatment tumor diameter and volume (Supplementary Figure, *r* = 0.853, *P* < 0.001, see online supplementary material). Figure 2B shows the relationship between EQD2 of the HR-CTV D90 and pretreatment tumor volume. Local recurrence was more frequent when the tumor volume was >90 cc and EQD2 was <70 Gy. Figure 2C displays the relationship between EQD2 of the HR-CTV D90 and the HR-CTV volume at first ICBT. Local recurrence was more frequent when the HR-CTV volume was ≥22 cc and EQD2 was <70 Gy. It was difficult to obtain EQD2 of the HR-CTV D90 ≥70 Gy when the HR-CTV volume was ≥22 cc. The relationship between EQD2 of the HR-CTV D90 and the distance from tandem to the lateral border of the HR-CTV is shown in Figure 2D. Local recurrence was more frequent when the distance to the lateral border of the HR-CTV was >2.5 cm and EQD2 was <70 Gy.

Table 4 summarizes the results of univariate and multivariate analyses. According to univariate analysis, the factors significantly associated with overall survival were the tumor histology (SCC vs non-SCC), HR-CTV D90 and use of four cycles or more of concurrent chemotherapy. Multivariate analysis indicated that HR-CTV D90 and concurrent chemotherapy were significant determinants of overall survival. In addition, univariate analysis showed that tumor size and HR-CTV D90 were factors significantly associated with local recurrence-free survival, while multivariate analysis only identified HR-CTV D90 as a significant determinant of local recurrence-free survival.

**Table 4:** Univariate and multivariate analyses results

	Overall survival			Local recurrence-free survival		
Parameter	HR	95% CI	*P* value^a^	HR	95% CI	*P* value
Univariate analysis						
Age (≧60 vs < 60)	1.113	0.635–1.950	0.708	1.583	0.787–3.185	0.198
Histology (SCC vs non- SCC)	2.651	1.048–6.706	0.040^*^	1.969	0.598–6.491	0.265
Hb (≦8 vs > 8)	2.047	0.955–4.388	0.065	1.386	0.486–3.957	0.541
SCC (≦10 vs > 10)	0.756	0.422–1.354	0.347	0.953	0.471–1.930	0.894
Tumor size (≧6 cm vs < 6 cm)	1.700	0.964–2.998	0.067	2.593	1.291–5.205	0.007^*^
Stage (I + II vs III + IV)	0.992	0.565–1.742	0.977	1.186	0.589–2.385	0.633
EQD2 HR-CTV D90						
(≧70 Gy vs < 70 Gy)	1.973	1.102–3.533	0.022^*^	4.180	1.804–9.686	<0.001^*^
OTT (<56 days vs ≧56 days)	1.411	0.721–2.762	0.315	1.448	0.650–3.226	0.365
Chemotherapy						
Neoadjuvant (yes vs no)	0.453	0.140–1.460	0.185	1.194	0.163–8.752	0.862
Concurrent (≧4 Cycles vs no)	0.499	0.279–0.895	0.020^*^	0.656	0.324–1.328	0.241
Adjuvant (yes vs no)	0.888	0.492–1.601	0.693	0.816	0.399–1.669	0.577
Multivariate analysis (stepwise method)						
EQD2 HR-CTV D90						
(≧70 Gy vs < 70 Gy)	2.015	1.125–3.608	0.019^*^	4.664	1.985–10.96	<0.001^*^
OTT (<56 days vs ≧56 days)			1.994	0.882–4.509	0.098	
Chemotherapy						
Concurrent (≧4 Cycles vs no)	0.489	0.273–0.877	0.016^*^			

^a^Cut off value for stepwise methods was set to *P* < 0.1. ^*^ Statistically significant (*P* < 0.05).

HR = Hazard ratio, CI = confidence interval, Hb = hemoglobin (g/dl), SCC = SCC antigen level (ng/ml), HR-CTV EQD2 = equivalent dose to 2 Gy of high risk CTV, OTT = overall treatment time.

**Fig. 3.**
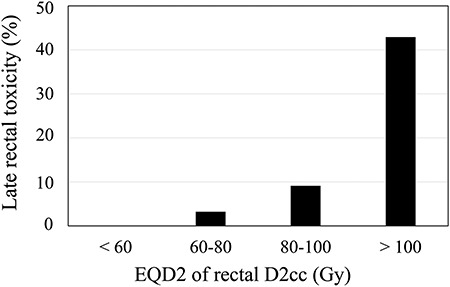
Relationship between grade 1 or higher late rectal toxicity and EQD2 of the rectal D2cc.

Late rectal toxicity occurred in 6 patients. Four patients had Grade 1 toxicity (transient bleeding), 1 patient had Grade 2 toxicity (bleeding requiring suppositories) and 1 patient had Grade 3 toxicity (rectal bleeding requiring blood transfusion and argon plasma laser coagulation). When the relationship between Grade 1 or higher late rectal toxicity and EQD2 of the rectal D2cc was investigated, the frequency of rectal toxicity gradually increased when EQD2 was >80 Gy (Fig. 3).

Late intestinal toxicity occurred in 5 patients. Three patients had Grade 3 toxicity (recurrent ileus due to radiation enterocolitis, ileus due to prior abdominal surgery and vesico-sigmoid fistula due to preceding pelvic infection) and 2 patients had Grade 4 toxicity (ileal perforation and sigmoid colon perforation potentially related to EBRT that required surgery). Four patients developed late genitourinary toxicity. Among them, 1 patient had Grade 1 toxicity (transient hematuria) and 3 patients had Grade 2 toxicity (hemorrhagic cystitis in 2 patients and ureteral stricture requiring a stent in 1 patient). There was no relationship between genitourinary toxicity and EQD2 of the bladder D2cc (84.2 Gy for Grade 1; and 85.9 Gy, 64.3 Gy and 54.7 Gy for Grade 2).

## DISCUSSION

In the present series, ICBT doses were prescribed for point A regardless of tumor size. However, ICBT dose prescription and evaluation using 3D ICBT planning parameters (i.e. HR-CTV D90, HR-CTV volume at initial ICBT, and the distance from the tandem to the lateral border of the HR-CTV) are essential for predicting local tumor control in cervical cancer patients receiving definitive radiotherapy.

This study demonstrated that local control of cervical cancer was significantly better when EQD2 of the HR-CTV D90 was ≥70 Gy than when it was <70 Gy (Figs 1D and 2A–C). To determine the EQD2 of the HR-CTV D90 dose for adequate local control, we reviewed four Japanese reports that are summarized in Table 5 together with our results. We should be careful when comparing these results, since different methods of prescribing brachytherapy were used (point A or HR-CTV D90). The authors from three institutions reported excellent local control rates with EQD2 of the HR-CTV D90 >60 Gy [5, 7, 8]. In addition, Okazaki *et al*. reported that EQD2 of the HR-CTV D90 >36 Gy was a significant predictor of local control by brachytherapy. In that study, the median whole pelvic dose was 30 Gy, so simple summation suggests that total EQD2 of the HR-CTV D90 >66 Gy may achieve better local control [9].

**Table 5:** Comparison of reported clinical outcomes of radiotherapy for uterine cervical cancer in Japan

Author	*n*	Type of IGBT	Pretreatment tumor size	HR-CTV vo lume at initial IGBT	EBRT Dose/fr	IGBT Dose/fr	LC rate					Findings
							I	II	Ill	IV	all	
Murakami *et al.* [5]	51	ICBT	I.8–7.7 cm	8.3—I 00.8 cc	WP20-50Gy	6 Gy/fr/point A	3 years					Better LC
National			median 4.5 cm	median 23.3 cc	(median 30Gy)	Total EQD2	NA	NA	NA	NA	90%	HR-CTV D90 < 60 Gy vs 60 Gy
Cancer Center						52.7–101.7 Gy (median 65.0 Gy)						
Ohno *et al.* [7]	80	ICBT66	< 4 cm 29	NA	WP 30 Gy + CS 20 Gy	IGBT 24 Gy/4 fr/HR-CTV D90	5 years					Better results with HR-CTV D90 > 60 Gy
Gunma		lCBT +needle 14	4–6 cm 34		WP 30–40 Gy + CS 10–20 Gy	HR-CTV D90 > 60Gy 90%	94%	97%	89% (lll	+lV)	94%	
Univ.			>6 cm 30		(Bulky case)							
Kusa da *et al.* [8]	68	IC BT 68	2.4–9.3 cm	IO—128 cc	WP 20-56Gy (median 40 Gy)	18 Gy/3 fr/Point A	2 years					OS and LC were signi ficant with
Ryukyu Univ.			median 4.6 cm	median 28 cc	CSNA Total 39.6–56 Gy		86%	83%	83%	50%	83%	HR-CTV D90 < 60 Gy vs > 60 Gy
					(median 50 Gy)							
Okazaki *et al*. [9]	103	ICBT94	< 4 cm 23	NA	WP 19.8–45 Gy	3–5 fr (median 4 fr)	2 years					HR-CTV D90 at BT >36Gy vs <36Gy
Saitama Medical		IC BT + IS BT 9	4–6 cm 50 > 6 cm 24		(median 30 Gy) CS 5.4–30 Gy	HR-CTV D90 25.9–55.9 Gy (median 40.5 Gy)	100%	96%	87% (lll	+IV)	NA	sig nificant for LC
Univ.					(median 20 Gy)	Total EQD2 50.4–90.9 Gy						
						(median 74.2 Gy)						
Current Study	89	ICBT 89	1. 5–11 cm	IO—108.7 cc	WP 30–50.4 Gy	1 2–24 Gy/2–4 fr/Point A	3 year					Better res ults with EQD2 HR-CTV
			median 5.2 cm	median 18.2 cc	(median 40 Gy)	Total EQD2 50.4–109.6 Gy	100%	66.5%	64.9%	68.6%	68.8%	D90 > 70 Gy, HR-CTV < 22 cc
			<3 cm 21		CS 3.6–22 Gy	(median 69.8 Gy)						Tandem to
			3–6 cm 53 > 6 cm 24		(median 12 Gy)							lateral border HR-CTV <2.5 cm

NA = Not assessed, WP = whole pelvis, CS = central shielding, OS = overall survival, BT = brachytherapy, LC = local control.

One explanation for the discrepancy between our results and the findings of these studies is the combination of the WP dose and IGBT dose. In three studies [5, 7, 9], patients mainly received 30 Gy to the WP and 24 Gy of IGBT in 4 fractions. However, only 10/89 patients (11%) received a similar protocol in our study, while the others received 40–50 Gy to the WP and 12–18 Gy of ICBT in 2–3 fractions. Therefore, our patients may not have fully benefited from targeting the high-dose area (e.g. the center of the primary tumor) by ICBT. Another possible explanation is that our patients may have had larger tumors and more advanced disease before treatment, since the median pretreatment tumor size was slightly larger in our study than in two of the other studies [5, 8] and a higher proportion of our patients had tumors larger than 6 cm compared to Ohno’s study (27.0 vs 21.3%) [7]. Accordingly, a higher EQD2 of the HR-CTV D90 might be required for local control when the tumor is larger.

As shown in Table 5, Okazaki *et al*. reported an excellent result of the 2-year local control rate of 87% for stage III + IV treated with IGBT, whereas our results show that the 3-year local control rates for stage III and IV treated with point A based ICBT are 64.9 and 68.6, respectively. These results indicate the need for the introduction of IGBT.

The Groupe Européen de Curiethérapie and European Society for Radiotherapy and Oncology (GEC-ESTRO) guidelines on 3D-IGBT for uterine cervical cancer and experts in this field recommend the use of T2-weighted MRI (T2WI) for delineating the target volume [1]. However, access to MRI may be limited in actual clinical practice, and most institutions in our country still utilize CT for 3D-IGBT. Although good treatment outcomes have been reported, CT-based 3D-IGBT achieves inferior soft tissue resolution compared to MRI-guided 3D-IGBT. Previous studies have demonstrated that the HR-CTV is consistently larger when contoured with CT than with MRI, especially in the lateral direction [10]. Since 2010, MRI has been routinely performed before initial ICBT at our hospital, and has been used as a reference for CT-guided ICBT. While MRI-guided IGBT is considered ideal, it can be difficult to implement due to lack of space for the MRI equipment and other problems.

Several factors can have an influence on the HR-CTV D90. We identified a negative correlation between HR-CTV D90 and the HR-CTV volume at initial ICBT (Fig. 2C), as well as between HR-CTV D90 and the distance from the tandem to the lateral border of the HR-CTV (Fig. 2D). Accordingly, a large HR-CTV volume could be one reason for a low HR-CTV D90 when ICBT doses are prescribed for point A. This suggests that dose escalation of EBRT with the aim of decreasing the HR-CTV is not a reasonable option. Instead, dose prescription based on HR-CTV D90 rather than point A is considered to be a better approach for delivering the appropriate dose to the entire tumor volume, especially when patients receive ICBT for large tumors. With this approach, simultaneous monitoring of doses to the OAR is mandatory [10–13]. The American Brachytherapy Society (ABS) guidelines suggest that the D2cc dose constraints are <75 Gy for the rectum/sigmoid and <90 Gy for the bladder [12]. In our study, 29 patients had a rectal D2cc >75 Gy and 15 patients had a bladder D2cc >90 Gy. There were no Grade 4 toxicities of the bladder or rectum, which was lower than the reports from the western series [3, 14]. A reason may be the use of central shielding in our study that decreases the actual cumulative rectal and bladder doses compared to the calculated doses in DVH analysis. When escalation of HR-CTV D90 over 70 Gy is attempted, attention must be paid to the rectal D2cc and the bladder D2cc in order to prevent severe complications affecting the rectum or bladder.

An asymmetrical tumor (i.e., marked parametrial invasion) increases the distance from the tandem to the lateral border of the HR-CTV, resulting in a lower EQD2 for the HR-CTV D90 and a higher risk of local recurrence. According to analysis of the retroEMBRACE study by Fokdal *et al*., when the HR-CTV volume was ≥30 cc, addition of Interstitial brachytherapy (ISBT) to ICBT improved the 3-year local control rate by 10% compared to ICBT alone [15]. In our study, when the HR-CTV volume was ≥22 cc, it was difficult to administer more than 70 Gy of EQD2 of the HR-CTV D90 with ICBT alone, and a higher local recurrence rate (66.7%) was observed (Fig. 2B).

Murakami *et al*. reported that progression-free survival was significantly better when the maximum distance from the tandem to the HR-CTV margin was <3.5 cm at first ICBT [5]. Kusada *et al*. also demonstrated that patients had a significantly inferior local control rate and disease-specific survival when the distance from the tandem to the lateral border of the HR-CTV was >3.0 cm [8]. In our series, a cut-off value of 2.5cm for the distance from the tandem to the HR-CTV border seemed to be an important prognostic indicator for local control (Fig. 2D). Taken together, these results suggest that the HR-CTV may not receive a sufficient dose if the distance from the tandem to the lateral border is >2 cm when the dose is prescribed for point A.

We used 40 Gy of EBRT instead of 30 Gy to obtain more tumor reduction and decrease the distance from the tandem to the lateral border of the HR-CTV. However, a 10 Gy increase of EBRT may not achieve sufficient tumor reduction and EQD2 of the HR-CTV D90 if the tumor diameter is ≥6 cm before treatment (Fig. 2A), if the tumor volume is ≥90 cc before treatment or ≥22 cc at the time of ICBT (Fig. 2B and C), or if the distance from the tandem to the HR-CTV border is >2.5 cm (Fig. 2D). Based on these results, for patients with larger tumors before initial ICBT, we should make an effort to administer a sufficient dose to the HR-CTV and avoid excessive irradiation of surrounding OAR during therapy by using a radioactive source dwell point and a time-optimization method such as graphical optimization and/or inverse planning simulated annealing. In cases where the optimization method is insufficient, interstitial or intra-tumoral insertion applicator should be considered.

In this study, 81 of 89 patients received CS EBRT and the CS width is shown in Table 2. Although the CS EBRT dose was excluded from our calculation of EQD2 of the HR-CTV D90, phantom studies have demonstrated that the contribution of CS EBRT to HR-CTV D90 should be considered and that it varies according to tumor size, shape and extent [16, 17]. However, most patients (82.7%) in our series had 4 cm of CS (Table 2) and the CS dose was small relative to the WP EBRT dose (90.7% of our patients received ≥39.6 Gy of WP EBRT), so variation of the CS dose and its effect on HR-CTV were limited compared to other reports.

In summary, we found that HR-CTV D90 was an important prognostic indicator for local recurrence. HR-CTV D90 >70 Gy was required for better local control, especially in patients with a larger HR-CTV (≥22 cc).

## Supplementary Material

Supplementary_figure_rrz099

## References

[1] Pötter R, Haie-Meder C, Van Limbergen E et al. Recommendations from gynaecological (GYN) GEC ESTRO working group (II): Concepts and terms in 3D image-based treatment planning in cervix cancer brachytherapy—3D dose volume parameters and aspects of 3D image-based anatomy, radiation physics, radiobiology. Radiother Oncol 2006;78:67–77. 16403584 10.1016/j.radonc.2005.11.014

[2] Dimopoulos JC, Pötter R, Lang S et al. Dose–effect relationship for local control of cervical cancer by magnetic resonance image–guided brachytherapy. Radiother Oncol 2009;93:311–5. 19679365 10.1016/j.radonc.2009.07.001

[3] Sturdza A, Pötter R, Fokdat LU et al. Image guided brachytherapy in locally advanced cervical cancer: Improved pelvic control and survival in RetroEMBRACE, a multicenter cohort study. Radiother Oncol 2016;120:428–33. 27134181 10.1016/j.radonc.2016.03.011

[4] Tanderup K, Fokdal LU, Sturdza A et al. Effect of tumor dose, volume and overall treatment time on local control after radiochemotherapy including MRI guided brachytherapy of locally advanced cervical cancer. Radiother Oncol 2016;120:441–6. 27350396 10.1016/j.radonc.2016.05.014

[5] Murakami N, Kasamatsu T, Wakita A et al. CT based three dimensional dose–volume evaluations for high-dose rate intracavitary brachytherapy for cervical cancer. BMC Cancer 2014;14:447. 24938757 10.1186/1471-2407-14-447 PMC4099086

[6] Ohno T, Wakatsuki M, Toita T et al. Recommendations for high-risk clinical target volume definition with computed tomography for three-dimensional image-guided brachytherapy in cervical cancer patients. J Radiat Res 2017;58:341–50. 27837120 10.1093/jrr/rrw109 PMC5440858

[7] Ohno T, Noda SE, Okonogi N et al. In-room computed tomography-based brachytherapy for uterine cervical cancer: Results of a 5-year retrospective study. J Radiat Res 2017;58:543–51. 27986859 10.1093/jrr/rrw121 PMC5766167

[8] Kusuda T, Toita T, Ariga T et al. Computed tomography–based image-guided brachytherapy for cervical cancer: Correlations between dose–volume parameters and clinical outcomes. J Radiat Res 2018;59:67–76. 29186565 10.1093/jrr/rrx065 PMC5778464

[9] Okazaki S, Murata K, Noda SE et al. Dose-volume parameters and local tumor control in cervical cancer treated with central-shielding external-beam radiotherapy and CT-based image-guided brachytherapy. J Radiat Res 2019;60:490–500. 31111896 10.1093/jrr/rrz023 PMC6640900

[10] Viswanathan AN, Beriwai S, De Los Santos JF et al. American brachytherapy society consensus guidelines for locally advanced carcinoma of the cervix. Part II: High dose-rate brachytherapy. Brachytherapy 2012;11:47–52. 22265437 10.1016/j.brachy.2011.07.002 PMC3489267

[11] Georg P, Pötter R, Georg D et al. Dose effect relationship for late side effects of the rectum and urinary bladder in magnetic resonance image–guided adaptive cervix cancer brachytherapy. Int J Radiat Oncol Biol Phys 2012;82:653–7. 21345618 10.1016/j.ijrobp.2010.12.029

[12] Mazeron R, Fokdal LU, Kirchheiner K et al. Dose–volume effect relationships for late rectal morbidity in patients treated with chemoradiation and MRI-guided adaptive brachytherapy for locally advanced cervical cancer: Results from the prospective multicenter EMBRACE study. Radiother Oncol 2016;120:412–9. 27396811 10.1016/j.radonc.2016.06.006

[13] Kato S, Tran DN, Ohno T et al. CT-based 3D dose–volume parameter of the rectum and late rectal complication in patients with cervical cancer treated with high-dose-rate intracavitary brachytherapy. J Radiat Res 2010;51:215–21. 20339256 10.1269/jrr.09118

[14] Zolciak-Siwinska A, Gruszczynska E, Bijok M et al. Computed tomography-planned high-dose-rate brachytherapy for treating uterine cervical cancer. Int J Radiat Oncol Biol Phys. 2016;96:87–92. 27375169 10.1016/j.ijrobp.2016.04.025

[15] Fokdal L, Sturdza A, Mazeron R et al. Image guided adaptive brachytherapy with combined intracavitary and interstitial technique improves the therapeutic ratio in locally advanced cervical cancer: Analysis from the retroEMBRACE study. Radiother Oncol 2016;120:434–40. 27113795 10.1016/j.radonc.2016.03.020

[16] Tamaki T, Ohno T, Noda SE et al. Filling the gap in central shielding: Three-dimensional analysis of the EQD2 dose in radiotherapy for cervical cancer with the central shielding technique. J Radiat Res 2015;56:804–10. 26062811 10.1093/jrr/rrv029 PMC4576998

[17] Tamaki T, Noda SE, Ohno T et al. Dose-volume histogram analysis of composite EQD2 dose distributions using the central shielding technique in cervical cancer radiotherapy. Brachytherapy 2016;15:598–606. 27475482 10.1016/j.brachy.2016.06.006

