# Supplementary figures and images for: Retrospective DVH analysis of point A based intracavitary brachytherapy for uterine cervical cancer
Source: J Radiat Res. 2020 Feb 1;61(2):265–74. doi: 10.1093/jrr/rrz099 (PMC7246069; doi:10.1093/jrr/rrz099)

# Supplementary figure

$r = 0.853, P < 0.001$

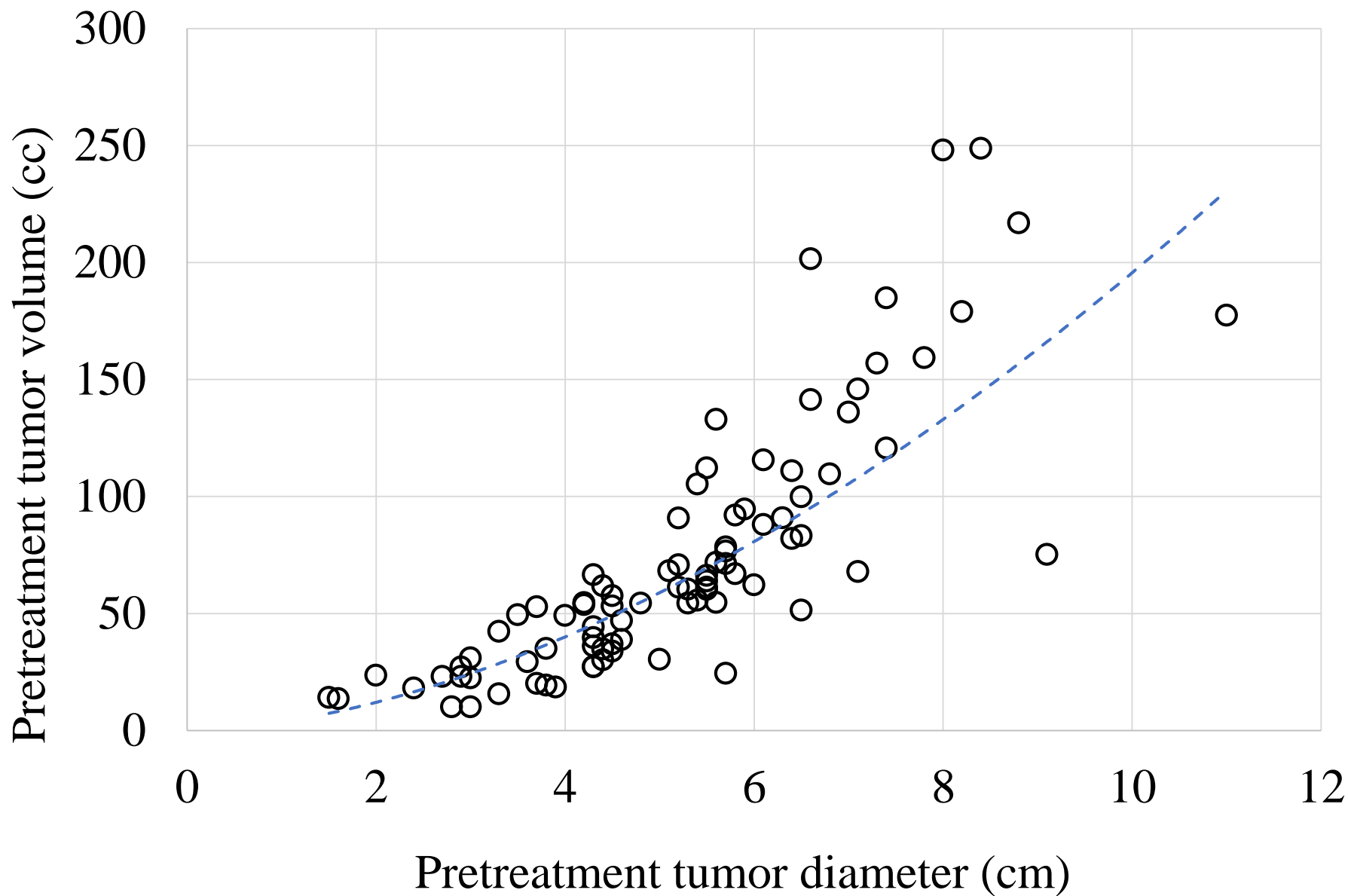

Supplement: Supplementary_figure_rrz099 [file supplementary_figure_rrz099.pdf]
